# Evaluation of Technology-Enhanced Learning Programs for Health Care Professionals: Systematic Review

**DOI:** 10.2196/jmir.9085

**Published:** 2018-04-11

**Authors:** Pam Nicoll, Sandra MacRury, Hugo C van Woerden, Keith Smyth

**Affiliations:** ^1^ National Health Service Education for Scotland Inverness United Kingdom; ^2^ University of the Highlands and Islands Inverness United Kingdom; ^3^ Cardiff University, Heathpark Campus Cardiff United Kingdom

**Keywords:** technology-enhanced learning, evaluation, e-learning, blended learning, digital learning, program evaluation, effectiveness

## Abstract

**Background:**

Technology-enhanced learning (TEL) programs are increasingly seen as the way in which education for health care professionals can be transformed, giving access to effective ongoing learning and training even where time or geographical barriers exist. Given the increasing emphasis on this mode of educational support for health care practitioners, it is vital that we can effectively evaluate and measure impact to ensure that TEL programs are effective and fit for purpose. This paper examines the current evidence base for the first time, in relation to the evaluation of TEL programs for health care professionals.

**Objective:**

We conducted a systematic review of the current literature relating to the evaluation of TEL programs for health care professionals and critically appraised the quality of the studies.

**Methods:**

This review employed specific search criteria to identify research studies that included evaluation of TEL for health care professionals. The databases searched included Medline Ovid, Cumulative Index of Nursing and Allied Health Literature Plus Advanced, Applied Social Sciences Index and Abstracts, ZETOC, Institute of Electrical and Electronics Engineers Explore Digital Library, Allied and Complementary Medicine, and Education Resources Information Center between January 2006 and January 2017. An additional hand search for relevant articles from reference lists was undertaken. Each of the studies identified was critically appraised for quality using the Crowe Critical Appraisal Tool. This approach produced a percentage total score for each study across specified categories. A proportion of the studies were independently assessed by an additional two reviewers.

**Results:**

The review identified 21 studies that met the inclusion criteria. The studies included scored totals across eight categories within a range of 37%-95% and an average score of 68%. Studies that measured TEL using learner satisfaction surveys, or combined pretest and posttest knowledge score testing with learner satisfaction surveys, were found to be the most common types of TEL evaluations evident in the literature. The studies reviewed had low scores across reporting on ethical matters, design, and data collection categories.

**Conclusions:**

There continues to be a need to develop effective and standard TEL evaluation tools, and good quality studies that describe effective evaluation of TEL education for health care professionals. Studies often fail to provide sufficient detail to support transferability or direct future TEL health care education programs.

## Introduction

The term technology-enhanced learning (TEL) is often used to describe a broad field of digital technologies used to support and mediate educational activities [1]. In this review, the term TEL is used to describe activities that are totally digitally mediated and those that are blended with more traditional educational approaches. The last two decades have seen considerable growth in the use of technology within higher education at undergraduate and postgraduate levels across the world [2]. Effective and ongoing continuous professional development (CPD) and education are essential to the delivery of high quality health and care services. TEL is increasingly presented as a means by which learners can be provided with enhanced or transformed educational experiences.

A range of published reports have highlighted TEL as an effective method to support health care education [3,4]. In their e-learning strategy, The Higher Education Funding Council England [5] summarized three levels of potential benefits of TEL: (1) e *fficiency*, whereby existing processes can be carried out in a more cost-effective, time-effective, sustainable, or scalable manner; (2) *enhancement*, which improves existing processes and outcomes; and (3) t *ransformation*, representing radical change in existing processes or the introduction of new processes. The recognition of the need for continuing education and effective work-based training to support health care professionals to deliver good quality, safe, and effective care is widely accepted [6,7]. The increasing demands for effective and affordable health care education in light of resource and time constraints, together with improved access to hardware, software, and the popularity of blended learning formats, means that TEL is increasingly considered an ideal approach within health care education.

The general availability of mobile and flexible technologies enables learners to minimize time away from health care settings to undertake training and to engage with learning resources when and where they are most suitable to their needs [8]. TEL offers a range of specific advantages for health care education, given the flexibility to update learning resources in a fast-changing field, and the scope offered for learners to share knowledge and learn critical clinical skills and decision making safely in nonclinical environments [9].

The ability to demonstrate the added value and impact of TEL for health care education remains challenging. Previous authors have captured the nature of the challenges in the review and evaluation of TEL within medical education [10-12]. Pickering and Joynes [13] highlighted the lack of robust evidence and meaningful evaluation to support widespread implementation of TEL resources. The main challenges concern a lack of clarity around the purpose of evaluation, comprehensiveness, depth, and methodology choice to support development of the required evidence base, upon which to build effective future TEL health care programs. We need reliable, practical mechanisms to evaluate: the value for money; equity of access; and learner, service, and organizational benefits that TEL may bring [14]. There is a need to critically examine the literature on TEL implementation and evaluation within health care to guide production and implementation of effective TEL health care education programs now and in the future.

A range of studies exist in the TEL literature which document implementation of TEL within medical and health care educational approaches. However, studies demonstrating a comprehensive TEL evaluation or use of standardized TEL evaluation tools in practice are fewer in number. Previous authors such as Ellaway [10,11], Cook and Ellaway [12], and Pickering and Joynes [13] (amongst others) have highlighted the need for robust evaluation, and have set out to develop both TEL educational standards and frameworks for evaluating TEL in medical education. Cook and Ellaway [12] have proposed a general model for evaluation. Pickering and Joynes [13] have proposed what they consider to be a more holistic TEL evaluation model for medical education.

This systematic review of the literature aimed to identify studies that have implemented TEL evaluation for CPD and postgraduate or work-based TEL health care education programs, and to assess these using a published critical appraisal tool. The studies identified provide an evidence base for the evaluation and development of future TEL programs for health care professionals.

## Methods

### Design

The review was carried out using a systematic integrative review method. This method allows for the inclusion of empirical and theoretical literature and quantitative and qualitative studies. This method enabled an increased number of studies to be included in the review and is appropriate for the review of evidence to highlight gaps in the literature [15].

### Eligibility and Inclusion

The inclusion and exclusion criteria for this review were developed using the Population, Intervention, Comparison, and Outcomes (PICOS) [16] framework for systematic reviews, which is illustrated in Table 1. In devising the search strategy for this study, the PICOS framework has been used as a search tool and as an organizing framework to list terms by the main concepts in the search question. This framework is commonly used to identify components of clinical evidence for systematic reviews in evidence-based medicine and is endorsed by the Cochrane Collaboration [16].

### Search Strategy

**Figure 1:**
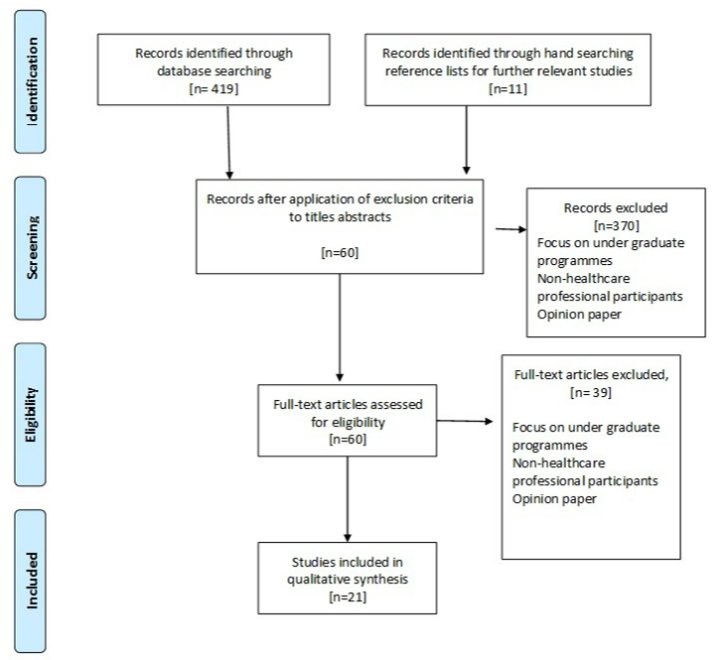
Literature search exclusion chart.

The following electronic databases were searched: Medline Ovid, Cumulative Index of Nursing and Allied Health Literature (CINAHL) Plus Advanced, Applied Social Sciences Index and Abstracts (ASSIA), ZETOC, Institute of Electrical and Electronics Engineers (IEEE) Explore Digital Library, Allied and Complementary Medicine (AMED), and Education Resources Information Center (ERIC) between January 2006 and January 2017 (see Multimedia Appendix 1 for details). The search was conducted using three concepts (and appropriate synonyms for each) across the selected databases: technology-enhanced learning, health care, and educational measurement. A total of 13 synonyms were used in the literature search. These phrases included technology-enhanced learning, technology-enhanced education, e-learning, e-education, blended learning, blended education, digital learning, digital education, evaluation studies, program evaluation, effectiveness, validation studies, and intervention. Reference lists were hand searched for relevant studies. The flow diagram in Figure 1 illustrates the search strategy.

**Table 1:** Application of the Population, Intervention, Comparison, and Outcomes (PICOS) Framework to the research question. TEL: technology-enhanced learning.

Parameter	Details
Participants	Health care professionals in full-time or part-time employment undertaking continuing professional development that is delivered using TEL (full time higher and further education students, school learners excluded).
Intervention	Studies using a TEL evaluation tool or framework to evaluate technology-enhanced health care education programs. The evaluation tool or framework must be used to evaluate a program for health care professionals.
Comparison	Some studies will have no comparison or comparator; others will examine one type of TEL approach against another.
Outcomes	Study must include: (1) evaluation of effective use of TEL, (2) the techniques being evaluated must be sufficiently specified, (3) assessment of learning outcomes, and (4) assessment of educational content.
Study design	Both empirical and theoretical research published in English between 2006-2017 from peer reviewed journals, conference papers. Systematic reviews and meta-analyses will not be included. Opinion papers will be excluded.

### Data Extraction

A specific data extraction tool was developed based on the inclusion and exclusion criteria. Each article was reviewed and information was extracted in relation to participant type, study design, sample size, types of TEL used, TEL evaluation tool used, and key study findings.

### Quality Assessment

Critical appraisal is a standardized way of assessing research so that decisions can be made based on the best evidence available [17]. The Crowe Critical Appraisal Tool (CCAT) was developed to provide a tool that can reliably assess a range of research designs, provide a comprehensive appraisal approach, and provide a suitable scoring method [17]. Quality assessment was conducted for all articles included within the study. The CCAT was used to assess quality across reporting items in eight categories: Preamble, Introduction, Design, Sampling, Data collection, Ethical matters, Results, and Discussion. The items were rated on a nominal scale (Present/Absent/Not applicable). The CCAT was selected as an appropriate critical appraisal tool for this study, as it can be reliably applied across a range of study designs and has been applied to a range of both quantitative and qualitative studies [17]. The CCAT emphasizes the importance of measuring and recording scores for each of the categories rather than simply the final score for each study. This approach prevents papers that score high overall, but poorly in one or more categories, from becoming less visible than papers that score highly throughout all categories. A subset of the studies [14,18-20] was randomly selected for scoring by additional reviewers (SM and HVW).

## Results

### Search Outcome

A total of 430 articles were identified in the initial titles search of the online databases and hand search of the literature. Following the application of the inclusion criteria to the titles and abstracts, 60 articles remained. The full text of the 60 articles were evaluated using the inclusion criteria and a total of 21 articles [14,18-37] were identified as suitable for the review.

Multimedia Appendix 2 shows a summary of the information extracted from the studies. Ten of the studies measured TEL using learner satisfaction surveys, 8 combined pretest and posttest knowledge score testing with learner satisfaction surveys (1 with pretest and posttest knowledge score testing only), and 2 used qualitative frameworks. The selected studies described the use of a variety of different TEL modes or combinations of each, including: 12 used Web-based e-learning, 4 used learning management systems, 2 used video simulation, and 6 used blended learning formats.

### Quality Assessment

The results of the quality assessment are summarized within Table 2. The CCAT scores for the studies selected using the inclusion criteria indicate that the quality of studies varied greatly across the range of research parameters examined. Of the 21 papers, 19 presented sufficient information to be included in the CCAT evaluation. Two papers [14,18] were identified as TEL articles rather than research papers with CCAT scores in early sections too low to be viable to continue scoring, in accordance with CCAT user guidance. Ten studies scored above the average score of 68% overall and 9 scored less than this average (these are italicized within Table 2). The lowest average-scored sections for the set of 19 studies were in Ethical Matters, with a score of 2/5. Design, Data Collection, and Results each produced average scores of 3/5. Preliminaries, Discussion, Introduction, and Sampling sections each produced the higher average scores of 4/5 for the studies examined.

These elements give a useful starting point in describing a requisite information set for inclusion within all good quality research studies. The value of studies that fail to include and compliment these basic elements with additional standard or sufficient research information data is significantly reduced. The studies examined had low scores for the Ethics section overall. This section looked for information related to consideration of standard research ethics, such as participant ethics and researcher ethics, even where formal ethical approval had not been required. Information that conveys ethical considerations is a prerequisite of all research studies.

The relatively low scores achieved in relation to Design, Data Collection, and Results categories are also concerning, as this renders many of the studies difficult (if not impossible) to replicate. This section looked for inclusion of information on interventions, outcomes, or treatment measures, in addition to sufficient descriptions of the research design and rationale. A key requisite of effective TEL evaluation research is ensuring that the intervention is sufficiently described to support others, who may wish to make comparisons, or to confidently apply the research to their own practice or education program development [11]. Only in this way will we begin to establish a reliable evidence base around TEL evaluation. The higher average quality assessment scores were in relation to the Discussion section and Preliminary and Introductory elements such as title, abstract, background, and objectives information.

A subset of the studies [14,18-20] was randomly selected for additional scoring by PN. The studies were provided to two of the coauthors of this paper (SM and HVW) for them to provide an additional score using the CCAT scoring template and guide. The average difference between the original and additional scoring across 4 of the papers was found to be 19%.

**Table 2:** Crowe Critical Appraisal Tool (CCAT) scores summary. Italics indicate studies that scored less than the average score of 68%. N/A: not applicable.

Authors	CCAT category								Raw score n (%)
	Preliminaries	Introduction	Design	Sampling	Data collection	Ethical matters	Results	Discussion	
Akroyd et al [14]	2	1	N/A	N/A	N/A	N/A	N/A	N/A	0
Lotrecchiano et al [18]	1	1	N/A	N/A	N/A	N/A	N/A	N/A	0
Westbrook [37]	2	4	1	1	3	0	1	3	15 (37.5)
*Konstantinidis et al [31]*	4	3	1	2	1	0	1	4	16 (40)
*Walsh et al [35]*	4	3	2	2	2	0	2	4	19 (47.5)
*Heartfield et al [29]*	2	4	2	2	2	1	3	4	20 (50)
*Chuo et al [24]*	3	4	3	3	4	0	2	3	21 (52.5)
*Wang [36]*	2	2	3	2	2	5	4	2	22 (55)
*Gill [27]*	3	4	4	2	3	2	3	4	23 (57.5)
*Ingelbeen et al [30]*	4	5	3	4	1	2	2	4	25 (62.5)
*Safwat and Pourabdollah [33]*	3	4	3	4	4	0	4	4	26 (65)
Goldberg Goetz et al [28]	5	5	3	5	4	0	3	5	30 (75)
Byrne et al [22]	3	5	4	4	4	4	4	4	32 (80)
Bekkers et al [21]	5	5	4	5	2	3	4	4	32 (80)
Popescu et al [32]	5	5	4	4	4	2	4	4	32 (80)
Sranacharoenpong et al [34]	5	5	4	5	5	1	4	3	32 (80)
Chang et al [23]	4	5	3	4	4	4	5	4	33 (82.5)
Moreira et al [19]	5	5	4	5	5	0	5	5	34 (85)
Fontaine et al [26]	5	5	3	5	5	2	5	5	35 (87.5)
Schneiderman et al [20]	5	5	4	4	4	4	4	5	35 (87.5)
Cortese-Peske [25]	5	5	4	5	4	5	5	5	38 (95)

## Discussion

### Principal Findings

Despite the growth in popularity and types of TEL education programs produced over the last two decades, this review was only able to identify a small pool of studies that met the inclusion criteria for TEL evaluation of a health care professional education program. Many of the included studies described the TEL methods evaluated as virtual learning environments, online or e-learning modules, platforms, or blended formats. There was little evidence provided within the selected studies regarding evaluation of bidirectional TEL approaches or newer types of TEL approaches such as Personal Learning Environments (PLEs). PLEs are activity spaces in which students interact and communicate with one another, and with experts, by using Web 2.0 tools. The ultimate result of using Web 2.0 tools is the development of collective learning approaches such as “just-in-time” and “at-your-fingertips” learning opportunities that can support a wide range of teaching and learning activities [38]. The evaluation studies identified in this review relied heavily on measuring TEL using learner satisfaction measures. Only one study [37] cited use of a structured approach to evaluating the more interactive two-way learning process between learner and tutor that is offered by models such as the Salmon-5 stage model [39]. The most widely cited types of learner outcome measurements used within educational evaluations are Kirkpatrick’s [40] models. While these methods of measurement may often provide a useful starting point for effective assessment of TEL evaluations, the model itself may not be ideally suited to the evaluation of TEL health care education. That is, where the Kirkpatrick model emphasizes increased confidence in newly acquired knowledge as being important, effective health care education would want evidence that this new knowledge is both learned and implemented in practice beyond a practitioner’s own perception of knowledge gain or confidence [13]. For health care education to truly support health care practice we need to be able to accurately measure the added knowledge, skills, or awareness that TEL programs may or may not provide.

This review of the literature concerning evaluations of TEL highlights the pattern that previous authors have noted for studies to employ a narrow focus in evaluation on either the technology equipment itself, measurement of learner satisfaction, or preknowledge and postknowledge scores [41,42]. Some of the studies included aspects of intralearner activity within their evaluation [18,22,27,28,30,33,37]. However, the dominance in the literature of evaluation studies measuring largely (or only) individual pretest and posttest knowledge presents a number of concerns within TEL for health care professionals. That is, where program assessments are relied upon to determine added value, learner gain, or improvement it is necessary to consider the extent to which they were matched to the actual TEL aims or enhancement being sought.

Testing methods can heavily influence the learner’s focus and how they approach learning. If we consider that one of the key functions of employing technology in learning is to help people connect more effectively with each other and the learning materials, and to inspire learner interaction in accordance with a social constructivist learning approach, then assessments that focus solely on the work of each individual (ie, cognitivist style, pretest and posttest scoring) may have a considerable impact on each learner’s behavior and the efficacy of the program overall [43]. There is an ongoing need for more TEL evaluation studies to detail the purpose of TEL interventions and the assessment and overall approaches adopted, and to demonstrate how the technology is enhancing the learning experience [2].

There is a need to be able to identify high quality TEL for health care education research studies and to be able to compare the outcomes from these sources to produce practical TEL evaluation tools. This need has also been highlighted throughout the last two decades in the literature on TEL in other contexts [44]. A more specific and standard approach to TEL evaluation in health care education with common measurements or tools would enable health care professionals, employers, and program designers to measure and then document effective TEL for health care education. In this way, a reliable TEL evidence base can grow and be progressively used to its full effect to influence or even transform educational programs for health care education now and in the future.

The CCAT tool used in this study enabled various forms of evidence presented within the literature to be explored in terms of TEL evaluation and the quality of the evidence presented. While some of the studies did present a full range of detailed research information, a number of those examined lacked information on the fundamental elements of good quality research. Ellaway [11] has highlighted similar issues related to published research for online learning where, as with TEL study interventions, they are often inadequately described. A more complex analysis of interassessor consistency in scoring using the CCAT tool could have been undertaken within this study. However, although the interassessor scoring was limited in this case by available resources, it was already building on the robust methods outlined by Crowe et al [17].

### Limitations

This review has not measured standard educational quality parameters or set out to identify the requisite elements of a robust TEL evaluation guide (or tool) for health care professionals’ education. Instead, it focused on identifying what evidence of TEL evaluation for health care professional education already exists within the literature and examined whether it was of sufficient quantity and quality as an evidence base for organizations to use to develop increasingly effective and transformative TEL education programs. Although the subject of TEL dates to at least the 1990s in the context of further and higher education, its application within health care education is much more recent. On that basis, the decision was made to restrict the literature search to 2006-2017. Other databases such as Web of Science were checked but yielded few references that fully met the inclusion criteria, and such databases were therefore not included within the literature review methods for this study.

### Conclusions

This review found limited published evidence of standard tools being implemented to measure TEL in health care education programs. Developing and implementing TEL health care education can require organizations to make considerable financial, human, and infrastructure investment. There is a mismatch between the scale of uptake of TEL in health care education and availability of a sufficiently robust evidence base of meaningful TEL evaluations in health care education. The outcomes of the systematic review and critical appraisal of this study support the views of Kirkwood and Price [2] who stated that there is a scarcity of published studies of practical TEL education programs that generate evidence that is appropriate to the interventions described, and that can be drawn upon.

A review of the TEL evaluation literature to help identify an evidence-based list of essential parameters to include within TEL health care education evaluation reports and studies would be a useful focus for further research. There continues to be a need to develop effective and standard TEL evaluation tools and for the publication of good quality studies that describe effective evaluation of TEL education for health care professionals **.** Studies often fail to provide sufficient detail to support transferability or direct future TEL health care education programs’ design and implementation. The use of a standard, practical, and quality approach to TEL evaluation, recording, and reporting with the same tools (or even parameters) across a variety of health care education programs would address this gap over time. This type of approach would also reduce duplication of efforts for organizations in creating or recreating tools, and importantly support cross-program and cross-organizational comparisons.

There is a range of guides, frameworks, and standards emerging in the literature and across practice to guide the design of TEL within health care and higher education institutions, programs, and resources. The models that have been proposed require widespread implementation, rigorous in-practice testing, and effective reporting to ensure that TEL education programs for health care professionals are evaluated in a more robust manner than is currently evident in the literature [13]. In this way, such programs can then usefully shape the emerging field of TEL evaluation for health care education.

## Appendix

Multimedia Appendix 1 Electronic database searches.

Multimedia Appendix 2 Summary of information from studies.

## Abbreviations

CCAT: Crowe Critical Appraisal Tool
CPD: continuous professional development
PICOS: Population, Intervention, Comparison, and Outcomes
PLE: Personal Learning Environment
TEL: technology-enhanced learning
