# Supplementary material for: Evaluation of Technology-Enhanced Learning Programs for Health Care Professionals: Systematic Review
Source: J Med Internet Res. 2018 Apr 11;20(4):e131. doi: 10.2196/jmir.9085 (PMC5917080; doi:10.2196/jmir.9085)
Supplement: Multimedia Appendix 1 [file jmir_v20i4e131_app1.pdf]

APPENDIX

Search Terms: 1& 3 first in non-health database then 2 if required

1 -Technology enhanced learning   2 – *Educational measurement   3 Health care

1	2	3
(technology enhanced learning or technology enhanced education).af.	*MESH Term +Medline filters<br>evaluation studies/ or evaluation studies as topic/ or program evaluation/ or validation studies as topic/ or ((pre- adj5 post-) or (pretest adj5 post-test) or (program* adj6 evaluat*)).ti,ab. or (effectiveness or [illegible]	health care.af.
(elearning or e-learning).af.		
e-education.af.		
(blended learning or blended education).af.		
(digital learning or digital education).af.		

Suggested database: Find 6 with an education and health/medical mix

Completed search 26th Oct *Ovid Medline/AMED/ CINAHL/ASSIA/ Zetoc/-

ACM Digital Library/Web of Science/IEE Explore/British Humanities Index

Additional search:
Google Scholar – advanced

Grey literature – government reports, national and international, European agencies reports

Ovid Medline

http://ovidsp.uk.ovid.com/sp-3.27.1a/ovidweb.cgi?&T=JS&PAGE=project&NAME=Ovid+26th+Oct+16

Owner: pam nicoll Created By: pam nicoll on 2016-Oct-26 12:12:09 PM
Search for: remove duplicates from 11 [limit 10 to (english language and yr="2006 - Current")]

Results: 70

Ovid MEDLINE(R) 1946 to Present with Daily Update			
**#**	**Search Statement**	**Results**	**Annotation**
1	(technology enhanced learning or technology enhanced education).af.	48	
2	(elearning or e-learning).af.	1473	
3	e-education.af.	53	
4	(blended learning or blended education).af.	270	
5	(digital learning or digital education).af.	38	
6	or/1-5	1785	
7	evaluation studies/ or evaluation studies as topic/ or program evaluation/ or validation studies as topic/ or ((pre- adj5 post-) or (pretest adj5 posttest) or (program* adj6 evaluat*)).ti,ab. or (effectiveness or intervention).ti,ab.	1046430	
8	6 and 7	615	
9	health care.af.	682369	
10	8 and 9	84	
11	limit 10 to (english language and yr="2006 -Current")	73	
12	remove duplicates from 11	70	

CINAHL Plus

Wednesday, October 05, 2016 10:40:07 AM

#	Query	Limiters/Expanders	Last Run Via	Results
S21	S16 AND S17	Limiters - Full Text<br>Search modes - Boolean/Phrase	Interface - EBSCOhost Research Databases<br>Search Screen - Advanced Search<br>Database - CINAHL Plus with Full Text	
S20	"technology enhanced education"	Search modes - Boolean/Phrase	Interface - EBSCOhost Research Databases<br>Search Screen - Basic Search<br>Database - CINAHL Plus with Full Text	Display
S19	"technology enhanced learning"	Search modes - Boolean/Phrase	Interface - EBSCOhost Research Databases<br>Search Screen - Basic Search<br>Database - CINAHL Plus with Full Text	Display
S18	S16 AND S17	Search modes - Boolean/Phrase	Interface - EBSCOhost Research Databases<br>Search Screen - Advanced Search<br>Database - CINAHL Plus with Full Text	
S17	(MM "Health+")	Search modes - Boolean/Phrase	Interface - EBSCOhost Research Databases	Display

			Search Screen - Basic Search<br>Database - CINAHL Plus with Full Text	
S16	S14 AND S15	Search modes - Boolean/Phrase	Interface - EBSCOhost Research Databases<br>Search Screen - Advanced Search<br>Database - CINAHL Plus with Full Text	
S15	"evaluation"	Limiters - Full Text; Published Date: 20060101-20141231<br>Search modes - Boolean/Phrase	Interface - EBSCOhost Research Databases<br>Search Screen - Basic Search<br>Database - CINAHL Plus with Full Text	Display
S14	S9 OR S10	Limiters - Full Text; Published Date: 20060101-20141231<br>Search modes - Boolean/Phrase	Interface - EBSCOhost Research Databases<br>Search Screen - Basic Search<br>Database - CINAHL Plus with Full Text	Display
S13	S9 OR S10	Limiters - Full Text<br>Search modes - Boolean/Phrase	Interface - EBSCOhost Research Databases<br>Search Screen - Basic Search<br>Database - CINAHL Plus with Full Text	Display
S12	S9 OR S10	Search modes - Boolean/Phrase	Interface - EBSCOhost Research Databases	Display

			Search Screen - Basic Search Database - CINAHL Plus with Full Text	
S11	S9 AND S10	Search modes - Boolean/Phrase	Interface - EBSCOhost Research Databases Search Screen - Basic Search Database - CINAHL Plus with Full Text	Display
S10	"digital learning"	Search modes - Boolean/Phrase	Interface - EBSCOhost Research Databases Search Screen - Basic Search Database - CINAHL Plus with Full Text	Display
S9	S1 OR S7	Search modes - Boolean/Phrase	Interface - EBSCOhost Research Databases Search Screen - Basic Search Database - CINAHL Plus with Full Text	Display
S8	S1 AND S7	Search modes - Boolean/Phrase	Interface - EBSCOhost Research Databases Search Screen - Basic Search Database - CINAHL Plus with Full Text	Display
S7	"e-education"	Search modes - Boolean/Phrase	Interface - EBSCOhost Research Databases	Display

			Search Screen - Basic Search Database - CINAHL Plus with Full Text	
S6	"e-education" AND (MH "Models, Educational") AND (MH "Education, Non-Traditional")	Search modes - Boolean/Phrase	Interface - EBSCOhost Research Databases Search Screen - Basic Search Database - CINAHL Plus with Full Text	Display
S5	S1 AND S2	Search modes - Boolean/Phrase	Interface - EBSCOhost Research Databases Search Screen - Basic Search Database - CINAHL Plus with Full Text	Display
S4	S1 AND S2	Search modes - Boolean/Phrase	Interface - EBSCOhost Research Databases Search Screen - Basic Search Database - CINAHL Plus with Full Text	Display
S3	S1 AND S2	Search modes - Boolean/Phrase	Interface - EBSCOhost Research Databases Search Screen - Basic Search Database - CINAHL Plus with Full Text	Display
S2	"technology enhanced education"	Search modes - Boolean/Phrase	Interface - EBSCOhost Research Databases	Display

			Search Screen - Basic Search Database - CINAHL Plus with Full Text	
S1	"technology enhanced learning"	Search modes - Boolean/Phrase	Interface - EBSCOhost Research Databases Search Screen - Basic Search Database - CINAHL Plus with Full Text	

ASSIA

(technology enhanced learning) AND (technology enhanced education OR technology enhanced education) AND (e-learning OR e-education) AND (blended learning OR blended education) AND (digital learning OR digital education) AND health AND (evaluation OR measurement) AND la.exact("English")
ZETOC
Additional limits - Date: From 2006 to 2016; Language: English

Results 38

- Source type
  - Scholarly Journals (34)
  - Dissertations & Theses (3)
  - Reports (1)

Zetoc Search > Search History

Search terms	Sort order	Options
any: health care date: 2006-2016 ti: evaluating blended learning		Edit
any: evaluation date: 2006-2016 ti: blended learning healthcare		Edit
any: evaluation date: 2006-2016 ti: blended learning	sorted on reverse date	View, Email Records, Download, Edit
any: evaluating date: 2006-2016 ti: technology enhanced learning	sorted on reverse date	View, Email Records, Download, Edit
any: healthcare date: 2006-2016 ti: technology enhanced learning	sorted on reverse date	View, Email Records, Download, Edit
any: health care date: 2006-2016 ti: technology enhanced learning		Edit

any: health care date: 2006-2016 ti: digital learning	sorted on reverse date	View, Email Records, Download, Edit
any: health care date: 2006-2016 ti: digital education evaluation		Edit
any: health care date: 2006-2016 ti: digital education	sorted on reverse date	View, Email Records, Download, Edit
any: health care date: 2006-2016 ti: blended learning	sorted on reverse date	View, Email Records, Download, Edit
date: 2006-2016 ti: blended learning healthcare	sorted on reverse date	View, Email Records, Download, Edit
date: 2006-2016 ti: blended learning	sorted on reverse date	View, Email Records, Download, Edit
date: 2006-2016 ti: evaluating digital education	sorted on reverse date	View, Email Records, Download, Edit
date: 2006-2016 ti: evaluating technology enhanced learning in healthcare		Edit
date: 2006-2016 ti: evaluating technology enhanced learning	sorted on reverse date	View, Email Records, Download, Edit
date: 2006-2016 ti: evaluatiing technology enhanced learning		Edit
date: 2006-2016 ti: evaluatiing technology enhanced learning in health care		Edit
date: 2006-2016 ti: technology enhanced learning	sorted on reverse date	View, Email Records, Download, Edit

AMED 26th Oct16

Search Results 8

1. (technology enhanced learning or technology enhanced education).af.
2. limit 1 to (full text and yr="2006 - 2016")
3. (elearning or e-learning).af.
4. e-education.af.
5. (blended learning or blended education).af.
6. (digital learning or digital education).af.
7. or/1-6
8. evaluation studies/ or evaluation studies as topic/ or program evaluation/ or validation studies as topic/ or ((pre- adj5 post-) or (pretest adj5 posttest) or (program* adj6 evaluat*)).ti,ab. or (effectiveness or intervention).ti,ab.
9. 7 and 8
10. health care.af.
11. 9 and 10
12. remove duplicates from 9

Search Name: ERIC 7.11.16

http://ovidsp.uk.ovid.com/sp-3.27.1a/ovidweb.cgi?&T=JS&PAGE=project&NAME=ERIC+9+7.11.16

Owner: pam nicoll

Created By: pam nicoll on 2016-Nov-07 03:13:49 PM

Last Modified By: pam nicoll on 2017-Nov-30 11:58:07 AM

Expires: 2017-Dec-31 11:58:07 AM

ERIC 7.11.16

1. (technology enhanced learning or technology enhanced education).af.
2. (elearning or e-learning).af.
3. e-education.af.
4. (blended learning or blended education).af.
5. (digital learning or digital education).af.
6. 1 or 2 or 3 or 4 or 5
7. evaluation studies/ or evaluation studies as topic/ or program evaluation/ or validation studies as topic/ or ((pre- adj5 post-) or (pretest adj5 posttest) or (program* adj6 evaluat*)).ti,ab. or (effectiveness or intervention).ti,ab.
8. 6 and 7
9. health care.af.
10. 8 and 9
11. limit 10 to (english language and yr="2006 - 2016")
12. remove duplicates from 11

IEE

Full Text Nov. 28, 2016 17:00 UTC

211 Results

(technology enhanced education") OR "technology enhanced learning") OR "elearning") OR "e-education") OR "blended learning") OR "blended education") OR "digital education") OR "digital learning") AND "health care") AND "evaluation")

matchBoolean%3Dtrue%26rowsPerPage%3D50%26searchField%3DSearch_All_Text%26queryText%3D%28%28%28%28%28%28%28%28%28%28.QT.technology+enhanced+education.QT.%29+OR+.QT.technology+enhanced+learning.QT.%29+OR+.QT.elearning.QT.%29+OR+.QT.e-education.QT.%29+OR+.QT.blended+learning.QT.%29+OR+.QT.blended+education.QT.%29+OR+.QT.digital+education.QT.%29+OR+.QT.digital+learning.QT.%29+AND+.QT.health+care.QT.%29+AND+.QT.evaluation.QT.%29
matchBoolean%3Dtrue%26searchField%3DSearch_All_Text%26queryText%3D%28%28%28%28%28%28%28%28%28%28.QT.technology+enhanced+education.QT.%29+OR+.QT.technology+enhanced+learning.QT.%29+OR+.QT.elearning.QT.%29+OR+.QT.e-education.QT.%29+OR+.QT.blended+learning.QT.%29+OR+.QT.blended+education.QT.%29+OR+.QT.digital+education.QT.%29+OR+.QT.digital+learning.QT.%29+AND+.QT.health+care.QT.%29+AND+.QT.evaluation.QT.%29 20 0
