# Supplementary material for: Evaluation of Technology-Enhanced Learning Programs for Health Care Professionals: Systematic Review
Source: J Med Internet Res. 2018 Apr 11;20(4):e131. doi: 10.2196/jmir.9085 (PMC5917080; doi:10.2196/jmir.9085)
Supplement: Multimedia Appendix 2 [file jmir_v20i4e131_app2.pptx]

## Slide 1
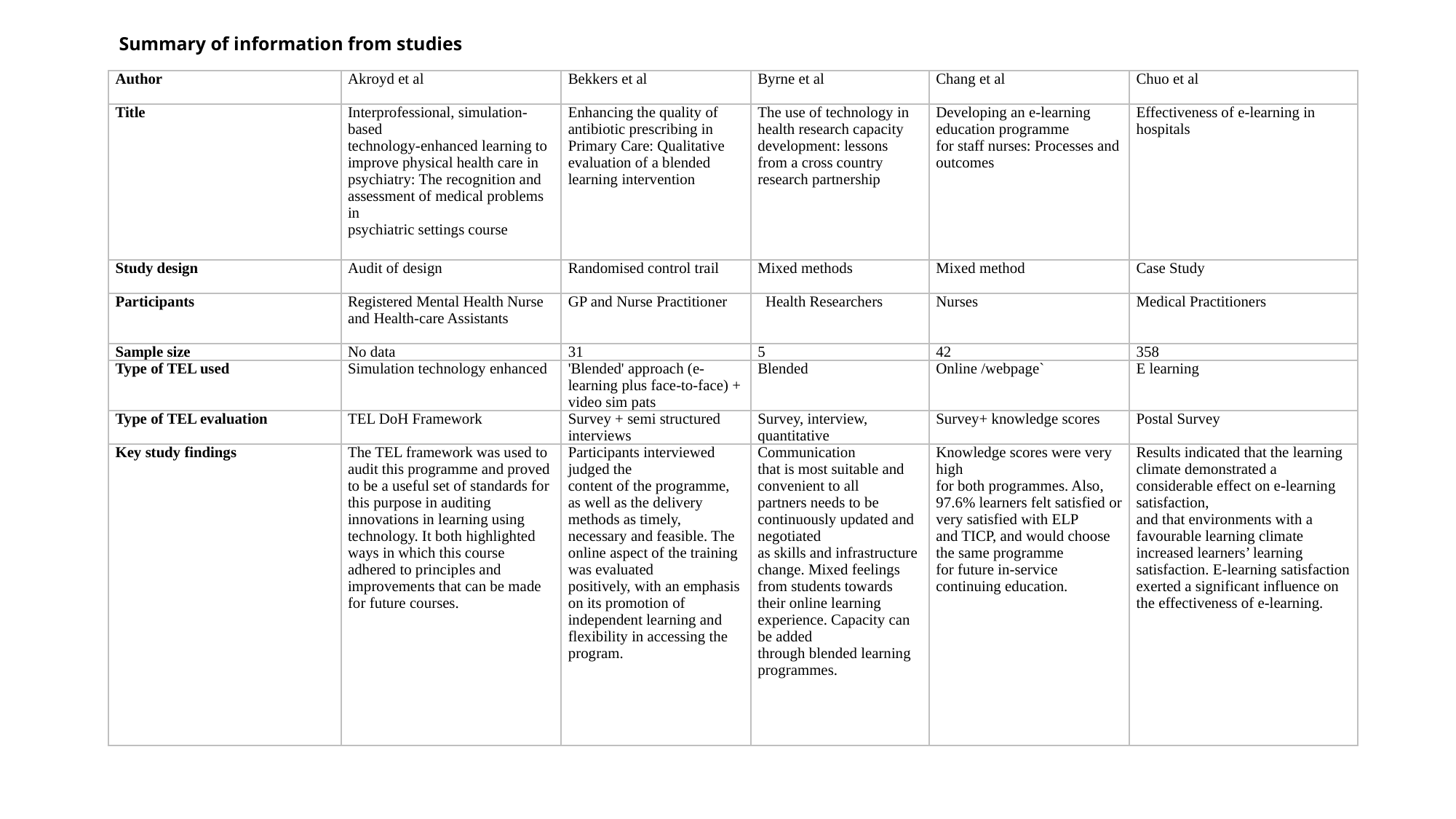

Summary of information from studies
| Author | Akroyd et al | Bekkers et al | Byrne et al | Chang et al | Chuo et al |
| --- | --- | --- | --- | --- | --- |
| Title | Interprofessional, simulation-based technology-enhanced learning to improve physical health care in psychiatry: The recognition and assessment of medical problems in psychiatric settings course | Enhancing the quality of antibiotic prescribing in Primary Care: Qualitative evaluation of a blended learning intervention | The use of technology in health research capacity development: lessons from a cross country research partnership | Developing an e-learning education programme for staff nurses: Processes and outcomes | Effectiveness of e-learning in hospitals |
| Study design | Audit of design | Randomised control trail | Mixed methods | Mixed method | Case Study |
| Participants | Registered Mental Health Nurse and Health-care Assistants | GP and Nurse Practitioner | Health Researchers | Nurses | Medical Practitioners |
| Sample size | No data | 31 | 5 | 42 | 358 |
| Type of TEL used | Simulation technology enhanced | 'Blended' approach (e-learning plus face-to-face) + video sim pats | Blended | Online /webpage` | E learning |
| Type of TEL evaluation | TEL DoH Framework | Survey + semi structured interviews | Survey, interview, quantitative | Survey+ knowledge scores | Postal Survey |
| Key study findings | The TEL framework was used to audit this programme and proved to be a useful set of standards for this purpose in auditing innovations in learning using technology. It both highlighted ways in which this course adhered to principles and improvements that can be made for future courses. | Participants interviewed judged the content of the programme, as well as the delivery methods as timely, necessary and feasible. The online aspect of the training was evaluated positively, with an emphasis on its promotion of independent learning and flexibility in accessing the program. | Communication that is most suitable and convenient to all partners needs to be continuously updated and negotiated as skills and infrastructure change. Mixed feelings from students towards their online learning experience. Capacity can be added through blended learning programmes. | Knowledge scores were very high for both programmes. Also, 97.6% learners felt satisfied or very satisfied with ELP and TICP, and would choose the same programme for future in-service continuing education. | Results indicated that the learning climate demonstrated a considerable effect on e-learning satisfaction, and that environments with a favourable learning climate increased learners’ learning satisfaction. E-learning satisfaction exerted a significant influence on the effectiveness of e-learning. |

## Slide 2
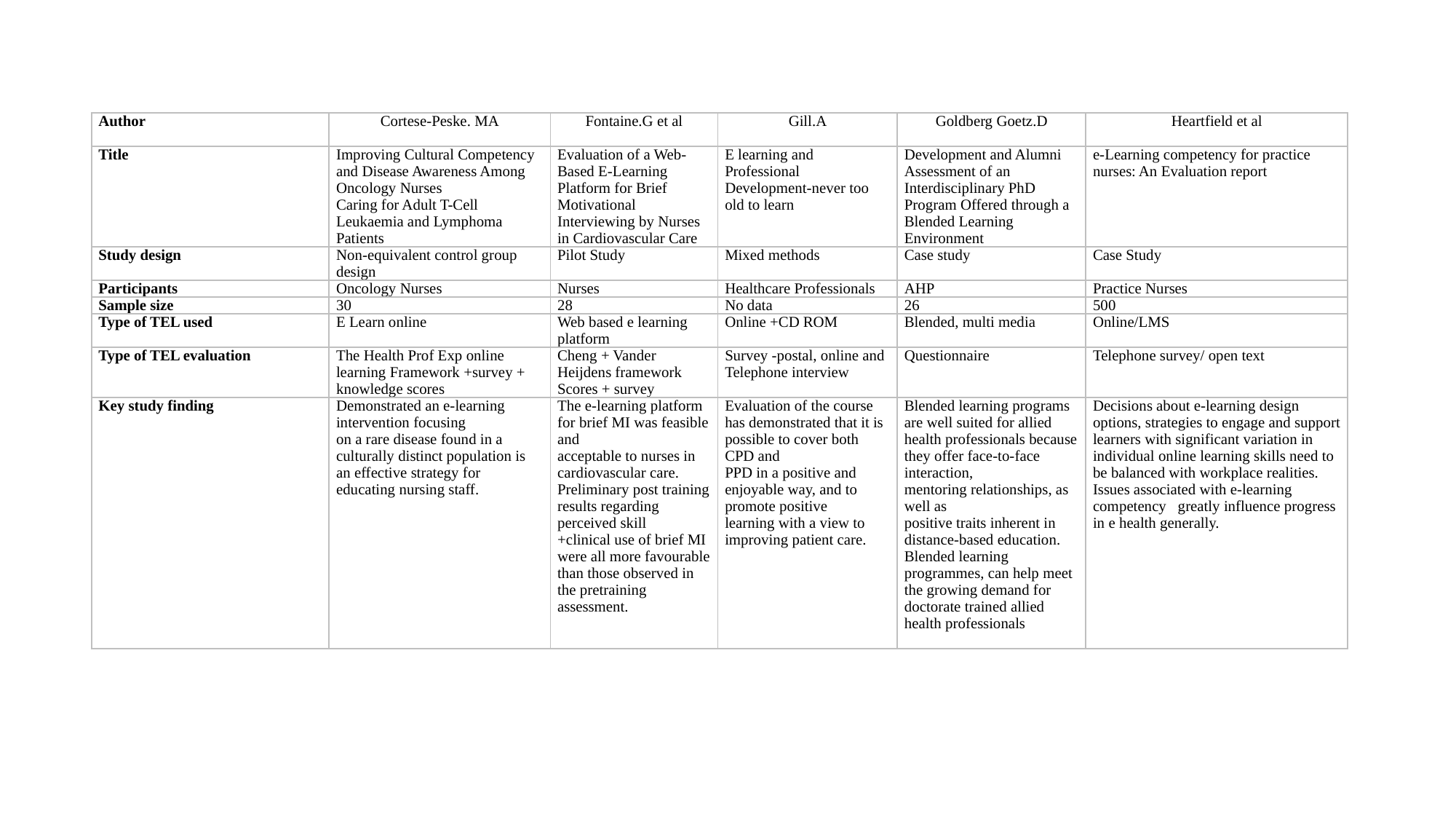

| Author | Cortese-Peske. MA | Fontaine.G et al | Gill.A | Goldberg Goetz.D | Heartfield et al |
| --- | --- | --- | --- | --- | --- |
| Title | Improving Cultural Competency and Disease Awareness Among Oncology Nurses Caring for Adult T-Cell Leukaemia and Lymphoma Patients | Evaluation of a Web-Based E-Learning Platform for Brief Motivational Interviewing by Nurses in Cardiovascular Care | E learning and Professional Development-never too old to learn | Development and Alumni Assessment of an Interdisciplinary PhD Program Offered through a Blended Learning Environment | e-Learning competency for practice nurses: An Evaluation report |
| Study design | Non-equivalent control group design | Pilot Study | Mixed methods | Case study | Case Study |
| Participants | Oncology Nurses | Nurses | Healthcare Professionals | AHP | Practice Nurses |
| Sample size | 30 | 28 | No data | 26 | 500 |
| Type of TEL used | E Learn online | Web based e learning platform | Online +CD ROM | Blended, multi media | Online/LMS |
| Type of TEL evaluation | The Health Prof Exp online learning Framework +survey + knowledge scores | Cheng + Vander Heijdens framework Scores + survey | Survey -postal, online and Telephone interview | Questionnaire | Telephone survey/ open text |
| Key study finding | Demonstrated an e-learning intervention focusing on a rare disease found in a culturally distinct population is an effective strategy for educating nursing staff. | The e-learning platform for brief MI was feasible and acceptable to nurses in cardiovascular care. Preliminary post training results regarding perceived skill +clinical use of brief MI were all more favourable than those observed in the pretraining assessment. | Evaluation of the course has demonstrated that it is possible to cover both CPD and PPD in a positive and enjoyable way, and to promote positive learning with a view to improving patient care. | Blended learning programs are well suited for allied health professionals because they offer face-to-face interaction, mentoring relationships, as well as positive traits inherent in distance-based education. Blended learning programmes, can help meet the growing demand for doctorate trained allied health professionals | Decisions about e-learning design options, strategies to engage and support learners with significant variation in individual online learning skills need to be balanced with workplace realities. Issues associated with e-learning competency greatly influence progress in e health generally. |

## Slide 3
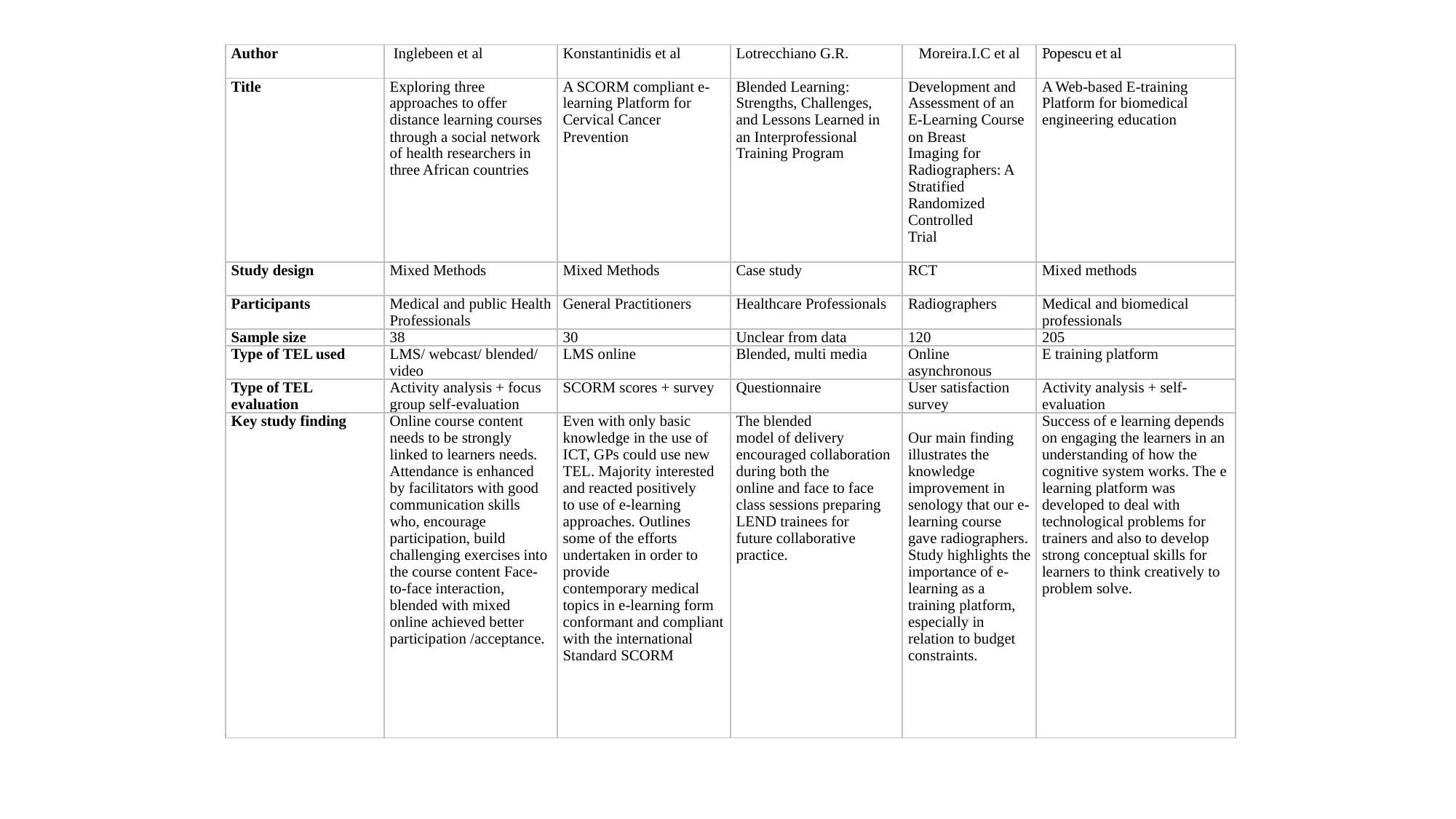

| Author | Inglebeen et al | Konstantinidis et al | Lotrecchiano G.R. | Moreira.I.C et al | Popescu et al |
| --- | --- | --- | --- | --- | --- |
| Title | Exploring three approaches to offer distance learning courses through a social network of health researchers in three African countries | A SCORM compliant e-learning Platform for Cervical Cancer Prevention | Blended Learning: Strengths, Challenges, and Lessons Learned in an Interprofessional Training Program | Development and Assessment of an E-Learning Course on Breast Imaging for Radiographers: A Stratified Randomized Controlled Trial | A Web-based E-training Platform for biomedical engineering education |
| Study design | Mixed Methods | Mixed Methods | Case study | RCT | Mixed methods |
| Participants | Medical and public Health Professionals | General Practitioners | Healthcare Professionals | Radiographers | Medical and biomedical professionals |
| Sample size | 38 | 30 | Unclear from data | 120 | 205 |
| Type of TEL used | LMS/ webcast/ blended/ video | LMS online | Blended, multi media | Online asynchronous | E training platform |
| Type of TEL evaluation | Activity analysis + focus group self-evaluation | SCORM scores + survey | Questionnaire | User satisfaction survey | Activity analysis + self-evaluation |
| Key study finding | Online course content needs to be strongly linked to learners needs. Attendance is enhanced by facilitators with good communication skills who, encourage participation, build challenging exercises into the course content Face-to-face interaction, blended with mixed online achieved better participation /acceptance. | Even with only basic knowledge in the use of ICT, GPs could use new TEL. Majority interested and reacted positively to use of e-learning approaches. Outlines some of the efforts undertaken in order to provide contemporary medical topics in e-learning form conformant and compliant with the international Standard SCORM | The blended model of delivery encouraged collaboration during both the online and face to face class sessions preparing LEND trainees for future collaborative practice. | Our main finding illustrates the knowledge improvement in senology that our e-learning course gave radiographers. Study highlights the importance of e-learning as a training platform, especially in relation to budget constraints. | Success of e learning depends on engaging the learners in an understanding of how the cognitive system works. The e learning platform was developed to deal with technological problems for trainers and also to develop strong conceptual skills for learners to think creatively to problem solve. |

## Slide 4
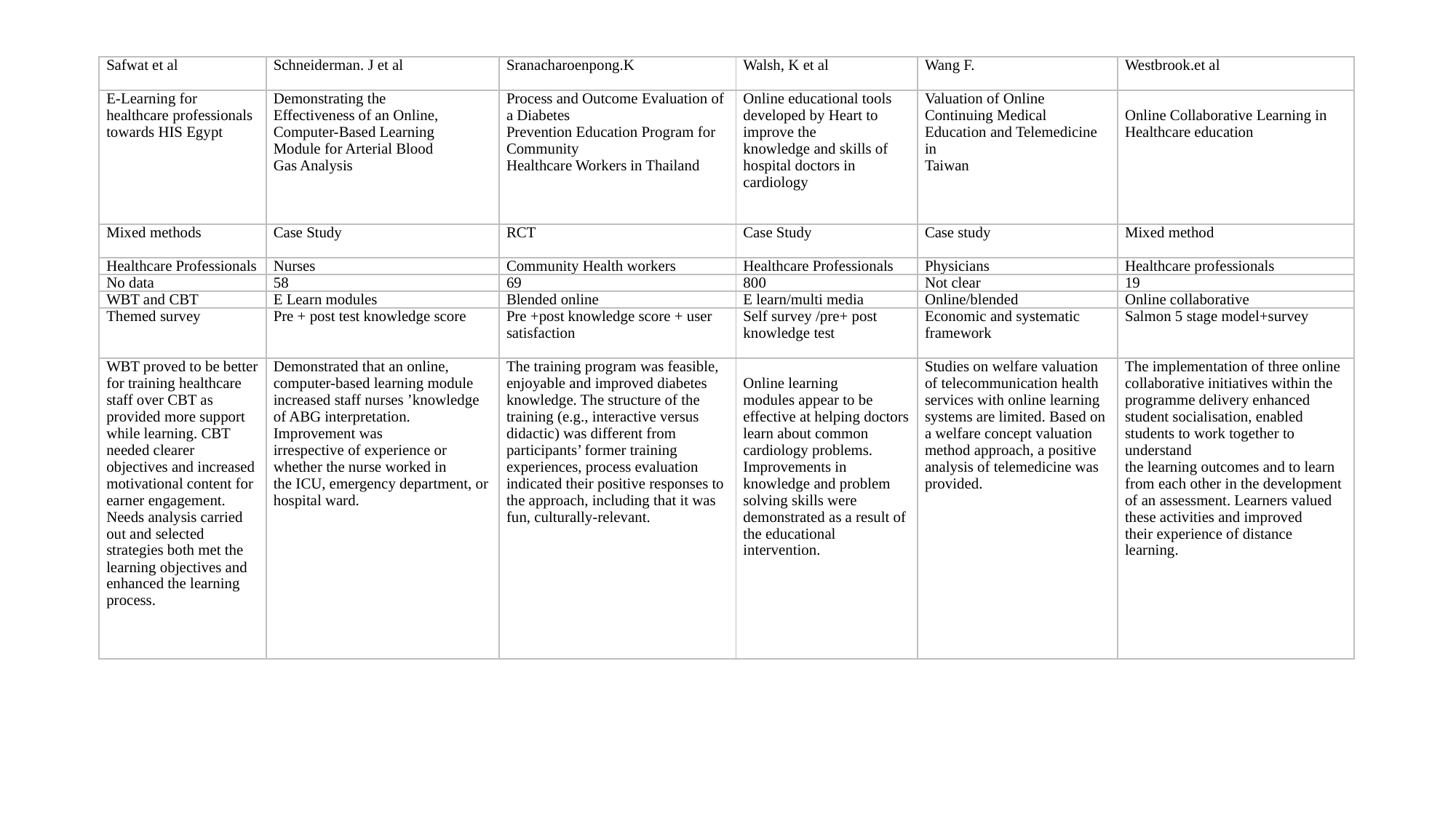

| Safwat et al | Schneiderman. J et al | Sranacharoenpong.K | Walsh, K et al | Wang F. | Westbrook.et al |
| --- | --- | --- | --- | --- | --- |
| E-Learning for healthcare professionals towards HIS Egypt | Demonstrating the Effectiveness of an Online, Computer-Based Learning Module for Arterial Blood Gas Analysis | Process and Outcome Evaluation of a Diabetes Prevention Education Program for Community Healthcare Workers in Thailand | Online educational tools developed by Heart to improve the knowledge and skills of hospital doctors in cardiology | Valuation of Online Continuing Medical Education and Telemedicine in Taiwan | Online Collaborative Learning in Healthcare education |
| Mixed methods | Case Study | RCT | Case Study | Case study | Mixed method |
| Healthcare Professionals | Nurses | Community Health workers | Healthcare Professionals | Physicians | Healthcare professionals |
| No data | 58 | 69 | 800 | Not clear | 19 |
| WBT and CBT | E Learn modules | Blended online | E learn/multi media | Online/blended | Online collaborative |
| Themed survey | Pre + post test knowledge score | Pre +post knowledge score + user satisfaction | Self survey /pre+ post knowledge test | Economic and systematic framework | Salmon 5 stage model+survey |
| WBT proved to be better for training healthcare staff over CBT as provided more support while learning. CBT needed clearer objectives and increased motivational content for earner engagement. Needs analysis carried out and selected strategies both met the learning objectives and enhanced the learning process. | Demonstrated that an online, computer-based learning module increased staff nurses ’knowledge of ABG interpretation. Improvement was irrespective of experience or whether the nurse worked in the ICU, emergency department, or hospital ward. | The training program was feasible, enjoyable and improved diabetes knowledge. The structure of the training (e.g., interactive versus didactic) was different from participants’ former training experiences, process evaluation indicated their positive responses to the approach, including that it was fun, culturally-relevant. | Online learning modules appear to be effective at helping doctors learn about common cardiology problems. Improvements in knowledge and problem solving skills were demonstrated as a result of the educational intervention. | Studies on welfare valuation of telecommunication health services with online learning systems are limited. Based on a welfare concept valuation method approach, a positive analysis of telemedicine was provided. | The implementation of three online collaborative initiatives within the programme delivery enhanced student socialisation, enabled students to work together to understand the learning outcomes and to learn from each other in the development of an assessment. Learners valued these activities and improved their experience of distance learning. |
